# Innovative Collaboration for a Longitudinal Cohort Study on the Health of Visually Impaired Individuals in Iran: A Partnership of NGOs, Private Entities, and Academia

**DOI:** 10.34172/ijhpm.9168

**Published:** 2025-10-07

**Authors:** Zahra Mortazavi, Keramat Yousofi, Shahriar Dabiri, Mohammad Khaksari

**Affiliations:** ^1^Independent Researcher, Kerman, Iran.; ^2^Clinical Research Development Unit, Mehrgan Hospital, Kerman University of Medical Sciences, Kerman, Iran.; ^3^Physiology Research Center, Institute of Neuropharmacology, Kerman University of Medical Sciences, Kerman, Iran.

## Dear Editor,

 We wish to share our experience launching a pioneering longitudinal cohort study in Iran, exemplifying a tripartite collaboration among academic, private, and non-governmental sectors. This initiative, titled “A 5-Year Prospective Cohort Study on the Physical, Mental, and Social Health of Individuals with Severe Visual Impairment and Blindness in Kerman, Iran,” represents a novel approach to addressing gaps in health research for vulnerable populations.

## Background and Rationale

 Globally, longitudinal studies focusing on the health of blind and severely visually impaired individuals remain scarce, particularly in low- and middle-income countries (LMICs).^1^ In LMICs, challenges such as limited funding, inadequate infrastructure, and low awareness of the issue hinder the effective development and implementation of programs. These factors make it difficult to establish sustainable solutions. In Iran, no comparable model exists to track the health trajectories of this population systematically. This absence makes it challenging to identify emerging health issues, allocate resources effectively, and design targeted interventions to reduce health disparities within this group. Existing research highlights significant unmet needs, including the progression of non-communicable diseases, mental health challenges (eg, depression, anxiety), and social determinants of health in visually impaired individuals.^2^ This cohort study aims to fill these knowledge gaps while testing a collaborative framework for multisectoral health research.

## Study Design and Collaborative Framework

 The study is a 5-year prospective cohort involving at least 700 participants registered with the Negahe Aftab Mehr Institute (a nonprofit supporting the visually impaired) and the Kerman Association of the Blind. The tripartite collaboration includes:

1. Academic Sector (Kerman University of Medical Sciences): Responsible for technical oversight, ethical governance, and partial financial support.
2. Private Sector (Mehrgan Hospital and Dr. Dabiri Diagnostic Laboratory): Provide clinical infrastructure and diagnostic services, contributing financially to the study.
3. Non-Governmental Organizations (NGOs) (Negahe Aftab Mehr Institute and Kerman Association of the Blind): Facilitate participant recruitment and community engagement and provide partial funding.

A memorandum of understanding formalizes the roles of this collaboration, including shared financial responsibilities, data collection, and dissemination of findings. This model ensures sustainability and equitable participation, addressing common challenges in LMIC-based research, such as fragmented funding and institutional silos (Figure).^3^

**Figure:**
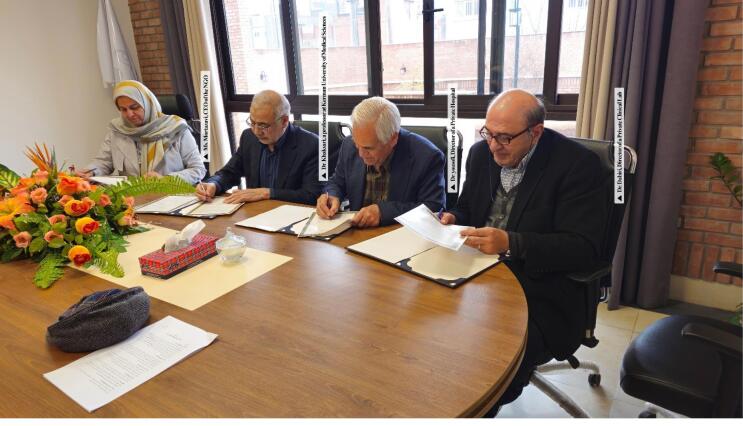
Signing of the Memorandum of Understanding Among Kerman University of Medical Sciences, the Private Sector, and Non-Governmental Organization, March 13, 2025.

## Innovation and Anticipated Outcomes

 This study introduces two key innovations. These innovations aim to bridge gaps in the research on the health of visually impaired individuals and have the potential to inform policies for better healthcare services.

 Furthermore, this study directly impacts the health of the target group by detecting health problems and facilitating medical care. Collected biobank samples will also be stored for potential future research or clinical use.

###  Methodological

 It is the first Iranian cohort to integrate physical, mental, and social health metrics for visually impaired individuals using validated tools.^4,5^

 We will measure demographic and anthropometric variables, general and psychological health, and lifestyle factors, including physical activity, sleep patterns, nutrition, smoking, alcohol and drug use, and medical history. An ophthalmologist will examine all participants and record key findings.^6-12^

 Simultaneously, a blood sample will be collected for routine lab tests, biobanking for future research, and assessment of specific biomarkers relevant to this group, such as melatonin, cortisol, thyroid and liver functions, and insulin levels. Subjects will receive a report containing interview results, lab tests, and physician records, and any abnormalities will be addressed.

 Annual assessments will track outcomes such as non-communicable disease progression, accident-related injuries, and mental health trends. The study will also assess vision trends in visually impaired subjects and ophthalmic complications in all subjects.

###  Operational

 This study benefited from a partnership in which the NGO provided the study site and equipment and recruited visually impaired participants. Private sector contributions included partial funding, technical support, lab tests, and participant incentives. Kerman Medical University covered additional study costs, addressed methodological concerns, and provided scientific and ethical approval of the research proposal.^13^

 Initial data (first-year data) will establish baseline health profiles. Subsequent years’ data will reveal health trends and their relationship to lifestyles and baseline risk factors. The archived biological samples provide a valuable resource for future research.

## Challenges and Lessons Learned

 Initially, coordination and establishing a shared language posed a significant challenge. However, an open dialogue platform enabled partners to comprehensively understand each other’s interests, capabilities, and limitations, culminating in a signed memorandum of understanding.^14^

## Conclusion and Call for Action

 Collaboration between academic, private, and NGO sectors offers an efficient model for organizing large, complex, and longitudinal studies, even in low-income countries. This cohort demonstrates the feasibility of such a multisectoral collaboration in resource-constrained settings, and we urge researchers and policy-makers to adopt similar frameworks to address health disparities in vulnerable groups.

## Acknowledgments

 We deeply appreciate the significant logistical and financial support provided by Negahe Aftab Mehr, a Kerman NGO supporting individuals with severe low vision and blindness. We also sincerely thank Dr. Dabiri Medical Lab, Mehregan Hospital, and Kerman University of Medical Sciences for their financial contributions to the project.

## Ethical issues

 This study has been approved by the Ethics Committee of Kerman University of Medical Sciences (IR.KMU.AH.REC.1403.159).

## Conflicts of interest

 Authors declare that they have no conflicts of interest.
